# Supplementary material for: Spatial spillover of local general higher education expenditures on sustainable regional economic growth: A spatial econometric analysis
Source: PLoS One. 2023 Nov 13;18(11):e0292781. doi: 10.1371/journal.pone.0292781 (PMC10642808; doi:10.1371/journal.pone.0292781)
Supplement: Attachment — Submitted filename: Response to reviewers.docx [file pone.0292781.s003.docx]

**Responses to Reviewer**

**Dear Reviewer,**

**Thank you for your comments concerning our manuscript “*Spatial spillover of local general higher education expenditures on sustainable regional economic growth: A spatial econometric analysis*”. First, we are grateful for the reviewer’s thoughtful comments and suggestions. Your valuable comments have helped us significantly improve our manuscript. We have thoroughly revised our manuscript according to the comments. The revised portions are marked in red in the article, and our responses and the reviewer’s original comments are given below in blue.**

**Reviewer**

*Comments to the Author(s)*

After careful consideration, we feel that it has merit but does not fully meet PLOS ONE’s publication criteria as it currently stands.

***Comment 1:*** Citations: Throughout the paper, we have noticed that there is a lack of appropriate citations to support your claims and arguments. We kindly request that you revisit your manuscript and make sure to cite relevant literature, including recent publications, to strengthen your arguments.

***Response 1:*** Thank you for your comments. We revisited the manuscript and cited relevant citations to support the arguments, including recent publications. For instance: Line 60, page 3; Line 68, page 4; Line 74, page 4; Line 91, page 5; Line 186, page 9; Line 215, page 11; Line 250, page 13; Line 290, 291, page 15; Line 302, page 16; Line 407, 410, page 23; Line 428, page 24; Line 485, page 27.

***Comment 2:*** Language and grammar: Proofread your manuscript for grammatical errors and awkward phrasing. This will improve the readability of your paper.

***Response 2:*** Thank you for your comments. We carefully checked the language and grammar of the manuscript, and it was deeply polished by AJE. Proof of polish has been submitted as an attachment.

***Comment 3:*** Structure and organization: Ensure that each section of your paper flows logically from one to the next. Make sure that you have clearly outlined your research questions, methods, results, and conclusions.

***Response 3:*** Thank you for your comments. Inspired by the reviewers' comments, we further adjusted the structure and organization. As mentioned in section 1 of the revised manuscript (Lines 112-116, page 6):

The paper is structured as follows. The following section reviews the related and latest research. Section 3 introduces the selection of variables and methods of data analysis. Section 4 reports the spatial characteristics of LGHEs and analyzes the effect of LGHEs on regional economic growth. Section 5 offers conclusions and further discussion. Section 6 discusses the limitations of the study and future work.

In addition, we add transitional language to ensure that each section of the manuscript flows logically from one to the next. For instance, Line 54, page3; Line 61, page3; Line 99, page 5; Line 220-221, page11; Line 429-430, page24; Line 482, page26.

Finally, we also further outline the outlined research questions, methods, results, and conclusions of our study. As mentioned in section 1 of the revised manuscript (Lines 71-111, pages 4-6):

The top priority of this study is to present the spatial characteristics of LGHEs. In describing the key features of regional differences, many scholars have used the Gini coefficient and the Theil index to quantitatively measure regional education expenditure disparities [8,9]. However, conventional statistical techniques have failed to distinguish the spatial differences. In this study, we investigated the spatial characteristics of LGHEs in China based on the exploratory spatial data analysis method (ESDA).

Another important purpose of this study is to explore the effect of LGHEs on regional economic growth. With the development of new economic geography, the role of education expenditure agglomeration in economic development is increasingly important [10]. However, most empirical studies that have investigated higher education expenditure (at its aggregate and disaggregate levels) and the economic growth nexus are based on the independence assumption and ignore the significant spatial interaction effects between the variables [11,12]. This may result in estimate bias [13], which may have led to an underestimation of the impacts of higher education expenditure on economic growth in the existing research. Therefore, we further apply the spatial Durbin model (SDM) to test the impact of LGHEs on regional economic growth.

To account for the spatial spillover effects, Anselin’s spatial theory was used as the theoretical basis for the spatial autocorrelation test, spatial econometric model selection and estimation [13]. Empirically, we investigated the spatial characteristics of LGHEs in China and tested the theoretical hypothesis that LGHEs can be influenced by neighboring provinces. Then, we used the SDM to calculate the direct and spillover effects of education expenditures on growth in different regions. Furthermore, the short- and long-term effects by which LGHEs promote economic growth were explored using the DSDM. An exploration of these issues is of great value for promoting educational equity and driving sustainable regional economic growth.

Different from previous studies, we offer two important contributions to the literature. First, we used ESDA to investigate the global and local spatial correlation characteristics of LGHEs. The ESDA method compensates for the defect of classical statistics that ignores geospatial elements and presents differences (spatial agglomeration or heterogeneity) in spatial distribution from a geospatial perspective [14,15]. Notably, LGHEs in a spatial agglomeration state are a premise for studying the spatial spillover of expenditures. Second, we built a more accurate SDM and dynamic spatial Durbin model (DSDM) with spatiotemporal fixed effects, which embrace spatially lagged terms, both dependent and independent variables, drawing on the complementary strengths of the spatial error model (SEM) and spatial lag panel (SLM) [13]. Since the literature concerning regional economic growth is rare, we used the models to investigate the direct and indirect effects and short- and long-term effects of LGHEs on the economic growth of different regions in China.

***Comment 4:*** Clarity of arguments: Ensure that your arguments are presented in a clear and logical manner and that each point is supported by appropriate evidence from the literature.

***Response 4:*** Thank you for your comments. We present the arguments in a clear and logical manner and cite relevant citations to support the arguments, including recent publications. For instance: Line 60, page 3; Line 68, page 4; Line 74, page 4; Line 91, page 5; Line 186, page 9; Line 215, page 11; Line 250, page 13; Line 290-291, page 15; Line 302, page 16; Line 407, 410, page 23; Line 428, page 24; Line 485, page 27.

***Comment 5:*** Discussion: The discussion section needs to be more focused on the implications of your findings. Consider discussing the broader context of your results and how they contribute to the existing body of knowledge. Be sure to address any limitations of your study and suggest avenues for future research.

***Response 5:*** Thank you for your comments. We revised the discussion section, further highlighted the contribution and significance of our study, and proposed relevant policy implications. In addition, we also address the limitations of the study and suggest avenues for future research. The suggestions are applicable not only for China but also for other countries. As mentioned in sections 5 and 6 of the revised manuscript (lines 467-496, pages 26-27):

Section 5: The contributions of this paper are as follows. First, we use EDSA to analyze the spatial characteristics of LGHE and verify the theoretical hypothesis that LGHEs have spatial autocorrelation. Second, spatial econometric analysis compensates for the ignorance of the spatial spillover effects of variables when exploring the relationship between the LGHE and regional economic growth. Finally, this paper expanded the application of spatial theory in the field of economic growth.

The findings are of great value to promoting fair investments in local general higher education, reducing regional disparities, and driving sustainable regional economic growth. They may also have significant implications worldwide for optimizing the allocation of scarce resources for education expenditures and the promotion of sustainable regional economic growth. In particular, our findings may provide useful suggestions for countries with regional economic growth disparities or that are experiencing the transformation of economic development patterns (e.g., from traditional to sustainable growth).

Section 6: There are still some limitations in this study. We focus on LGHEs as the independent variable and do not consider the influence of other educational factors (e.g., per student schooling years) on economic growth. In fact, human capital and physical capital jointly affect economic growth [18]. Therefore, educational factors should be considered comprehensively to improve the scientific rationality of measuring the impact of local general higher education on economic growth. This study also lacks comparative studies across multiple countries.

In the future, there is still much pertinent work to do. For instance, the influence of changes in higher education expenditure inequality on economic growth should be discussed across multiple countries. Future work may also explore how spillover effects of other educational factors (e.g., per student schooling years) affect the sustainable growth of regional economies. Researchers can further expand the analysis of regional spillover effects from one province to another without using regions as boundaries to illustrate the reason and path for the spatial spillover effects of educational factors.

***Comment 6:*** Figures and tables: Ensure that all figures and tables are clear, properly labeled, and referenced within the text. Additionally, provide more detailed captions to help readers understand the information presented.

***Response 6:*** Thank you for your comments. We have improved the format of all figures using Preflight Analysis and Conversion Engine (PACE) digital diagnostic tool and tables to ensure that they are clear, properly labeled, and referenced within the text. In addition, we provide more detailed captions. For instance: Line 262, page 14; Line 284, page 15; Line 306, page 16; Line 312, page17; Line 342, page 19; Line 365, page 20; Line 389, page 22. Finally, we drew the LISA cluster diagram of LGHEs (Fig 1), and further revise the figure using Preflight Analysis and Conversion Engine (PACE) digital diagnostic tool. As mentioned in section 4 of the revised manuscript (Line 283, page 15):

**Fig 1. LISA clustering results of LGHEs.** a. 2000, b. 2007, c. 2014, d. 2021.

**References:**

8. Akita, T. Educational expansion and the role of education in expenditure inequality in indonesia since the 1997 financial crisis. Social Indicators Research. 2017; 130(3):1165–1186. https://doi.org/10.1007/s11205-015-1208-y
9. Varughese AR, Bairagya I. Interstate variation in household spending on education in India: Does it influence educational status?. Structural Change and Economic Dynamics. 2021; 59:405–415. <https://doi.org/10.1016/j.strueco.2021.09.015>
10. LeSage JP, Pace RK. Introduction of spatial econometrics. 1st ed. Boca Raton: CRC Press; 2009.
11. Ojha VP, Ghosh J, Pradhan BK. The role of public expenditure on secondary and higher education for achieving inclusive growth in India. Metroeconomica. 2022; 73(1):49–77. https://doi.org/10.1111/meca.12353
12. Zhu TT, Peng HR, Zhang YJ. The influence of higher education development on economic growth: Evidence from central China. Higher Education Policy. 2018; 31(2):139–157. https://doi.org/10.1057/s41307–017–0047–7
13. Anselin L. Thirty years of spatial econometrics. Papers in Regional Science. 2010; 89(1):3–25. <https://doi.org/10.1111/j.1435–5957.2010.00279.x>
14. Wang F, Chai W, Shi XT, Dong MR, Yan B. Does regional financial resource contribute to economic growth? From the perspective of spatial correlation network. SAGE Open. 2021; 11(1). https://doi.org/10.1177/2158244021999381
15. Ma SJ, Li L, Ke HM, Zheng YL. Environmental protection, industrial structure and urbanization: Spatiotemporal evidence from Beijing–Tianjin–Hebei, China. Sustainability. 2022; 14(2). https://doi.org/10.3390/su14020795
18. Ding S, Knight J. Why has China grown so fast? The role of physical and human capital formation. Oxford Bulletin of Economics and Statistics. 2011; 73(2):141–174. https://doi.org/10.1111/j.1468–0084.2010.00625.x

*We earnestly appreciate your warm work, and we offer special thanks to you for your valuable comments.*

*We tried our best to improve the original manuscript and made significant changes in the revised manuscript. These changes will not influence the content of the article. Here, we earnestly appreciate the reviewer’s warm work and hope that the connection will meet with approval. In the meantime, we appreciate the editor’s invitation to modify our manuscript. Thank you for giving us the chance to submit the revised manuscript.*

*Once again, thank you very much for your comments and suggestions.*

*Best wishes,*

*Authors of this article*
