# Supplementary material for: Spatial spillover of local general higher education expenditures on sustainable regional economic growth: A spatial econometric analysis
Source: PLoS One. 2023 Nov 13;18(11):e0292781. doi: 10.1371/journal.pone.0292781 (PMC10642808; doi:10.1371/journal.pone.0292781)
Supplement: Attachment — Submitted filename: Responses to Reviewers.docx [file pone.0292781.s004.docx]

**Responses to Reviewer #1**

**Dear Reviewer,**

**Thank you for your comments concerning our manuscript “*Spatial spillover of local general higher education expenditures on sustainable regional economic growth: A spatial econometric analysis*”. First, we are grateful for the reviewer’s thoughtful comments and suggestions. Your valuable comments have helped us significantly improve our manuscript. We have thoroughly revised our manuscript according to the comments. The revised portions are marked in red in the article, and our responses and the reviewer’s original comments are given below in blue.**

**Journal Requirements**

Please review your reference list to ensure that it is complete and correct. If you have cited papers that have been retracted, please include the rationale for doing so in the manuscript text, or remove these references and replace them with relevant current references. Any changes to the reference list should be mentioned in the rebuttal letter that accompanies your revised manuscript. If you need to cite a retracted article, indicate the article’s retracted status in the References list and also include a citation and full reference for the retraction notice.

***Response:*** Thank you for your comments. We reviewed the reference list and ensured that it was complete and correct. We do not cite papers that have been retracted. Any changes to the reference list have been mentioned in the rebuttal letter that accompanies the revised manuscript. For instance, Lines 540, 544, 546, 550, page 30; Lines 568, 579, page 31; Lines 583, 599, page 32; Lines 603, 606, page 33; Lines 623, 632-637, page 34; Line 683, page 36; Lines 696-697, 703, page 37; Line 706, page 37; Line 718, page 38; Line 732, page 39.

**Reviewer #1**

*Comments to the Author(s)*

Overall, your manuscript has the potential to make a valuable contribution to the field. By addressing the above-mentioned points, you can significantly improve the clarity, rigor, and impact of your study.

***Comment 1:*** Firstly, I would like to suggest improving the literature review section by incorporating relevant papers. Specifically, the following papers may provide valuable insights and enhance the overall quality of your manuscript:

1. Bao, Z., & Huang, D. (2020). "Gender differences in reaction to enforcement mechanisms: A large-scale natural field experiment." This paper explores gender differences in response to enforcement mechanisms, which could be relevant to understanding the impact of education expenditures on regional economic growth. By considering potential gender-specific effects, you can provide a more comprehensive analysis of the relationship between education expenditures and economic outcomes.

2. Chen, M., Huang, D., & Wu, B. (2022). "Interlocking Directorates and Firm Performance: Evidence from China." This study investigates the relationship between interlocking directorates and firm performance, which could be relevant to understanding the mechanisms through which education expenditures influence regional economic growth. By considering the role of corporate governance and inter-firm connections, you can provide a more nuanced analysis of the impact of education expenditures.

Incorporating these papers into your literature review will help situate your study within the existing literature and provide a more comprehensive understanding of the topic.

***Response 1:*** Thank you for your comments. Accordingly, we incorporated the abovementioned papers into the literature review section. By considering potential gender-specific effects and the role of corporate governance and interfirm connections, we provide a more comprehensive analysis of the relationship between education expenditures and economic outcomes. As mentioned in Section 2 and reference section of the revised manuscript (Lines 141-144, page 7; Lines 632-637, page 34):

Furthermore, its economic growth effects are affected by gender differences in reactions to enforcement messages, as well as by governance structures and interorganizational connections [32,33].

References:

32. Bao ZY, Huang DF. Gender differences in reactions to enforcement mechanisms: A large-scale natural field experiment; 2020 [cited 2023 Sep 26]. Database: SSRN [Internet]. Available from: http://dx.doi.org/10.2139/ssrn.3641282
33. Chen MZ, Huang DF, Wu BY. Interlocking Directorates and Firm Performance: Evidence from China; 2022 [cited 2023 Sep 26]. Database: SSRN [Internet]. Available from: <http://dx.doi.org/10.2139/ssrn.4005022>

***Comment 2:*** Clarify the research question and objectives: It would be helpful to explicitly state the research question and objectives of your study. This will provide a clear roadmap for the readers and help them understand the purpose and significance of your research.

***Response 2:*** Thank you for your comments. Based on the research background, we propose research questions and further clarify the research objectives of this study. The research questions in this study are as follows: What kind of spatial characteristics do LGHEs present? What impact do LGHEs have on regional economic growth, and how does it affect regional growth? The research objectives of this study are as follows: The top goal of this study is to present the spatial characteristics of LGHEs. Another important purpose of this study is to explore the effect of LGHEs on regional economic growth. As mentioned in Section 1 of the revised manuscript (Lines 69-91, pages 4-5):

Hence, it is necessary to explore the following questions in this study: What kind of spatial characteristics do LGHEs present? What impact does LGHEs have on regional economic growth, and how does it affect regional growth? These issues are important for promoting educational equity and driving sustainable regional economic growth.

The top goal of this study is to present the spatial characteristics of LGHEs. In describing the key features of regional differences, many scholars have used the Gini coefficient and the Theil index to quantitatively measure regional education expenditure disparities [8,9]. However, conventional statistical techniques have failed to distinguish the spatial differences. In this study, we investigated the spatial characteristics of LGHEs in China based on the exploratory spatial data analysis method (ESDA).

Another important purpose of this study is to explore the effect of LGHEs on regional economic growth. With the development of new economic geography, the role of education expenditure agglomeration in economic development is increasingly important [10]. However, most empirical studies that have investigated higher education expenditure (at its aggregate and disaggregate levels) and the economic growth nexus are based on the independence assumption and ignore the significant spatial interaction effects between the variables [11,12]. This may result in estimate bias [13], which may have led to an underestimation of the impacts of higher education expenditure on economic growth in the existing research. Therefore, we further apply the spatial Durbin model (SDM) to test the impact of LGHEs on regional economic growth.

***Comment 3:*** Provide more details on the methodology: While you have mentioned the use of spatial theory and the spatial Durbin model, it would be beneficial to provide more details on the specific methods and techniques employed. This will enable readers to better understand the analytical approach and replicate your study if desired.

***Response 3:*** Thank you for your comments. We provide more details on spatial theory. Anselin’s spatial theory constitutes a comprehensive analysis of spatial econometrics, and we use Anselin’s spatial theory as the theoretical basis for the spatial autocorrelation test, spatial econometric model selection and estimation in the study. As mentioned in Section 1 of the revised manuscript (Lines 92-96, page 5):

As a pioneer of spatial econometrics research, Anselin (2010) put forward his spatial theory, which constitutes a comprehensive analysis of spatial econometrics [13]. To account for the spatial spillover effects, Anselin’s spatial theory was used as the theoretical basis for the spatial autocorrelation test, spatial econometric model selection and estimation.

Furthermore, we provide more details on the use of the spatial Durbin model (SDM) and dynamic spatial Durbin model (DSDM). As mentioned in Section 3 and reference section of the revised manuscript (Lines 253-262, pages 13-14; Lines 267-271, page 14; Lines 696-697, page 37):

SDM:

If there are spatial lag terms, the regression coefficient of the econometric model cannot directly be used to reflect the marginal effect of independent variables. To judge the spatial spillover effect of LGHEs on regional economic growth more effectively, this study applies the partial differential method to measure the direct and spillover effects in the SDM [10,52], accounting for the spatial correlation information of the dependent variable and the independent variable. Equations 5-7 show the direct, spillover, and total effects of the SDM.

|  | $\text{Direct effects=}\left[\left(I_{N}-\rho W\right)^{-1}\left(\beta_{k}I_{N}+\theta_{\text{k}}W\right)\right]^{\overline{d}}$ | (5) |
| --- | --- | --- |
|  | $Spillover\text{ effects=}\left[\left(I_{N}-\rho W\right)^{-1}\left(\beta_{k}I_{N}+\theta_{\text{k}}W\right)\right]^{\overline{rsum}}$ | (6) |
|  | $\text{Total effects=}\left[\left(I_{N}-\rho W\right)^{-1}\left(\beta_{k}I_{N}+\theta_{\text{k}}W\right)\right]^{\overline{d}}+\left[\left(I_{N}-\rho W\right)^{-1}\left(\beta_{k}I_{N}+\theta_{\text{k}}W\right)\right]^{\overline{rsum}}$ | (7) |

where I_N_ is an identity matrix, k represents k-th explanatory variable, and ‾d and‾rsum denote two operators that can be used to calculate both the mean diagonal element and the mean row sum of the nondiagonal elements of a matrix [52].

Reference:

52. Elhorst JP. Matlab Software for Spatial Panels. International Regional Science Review. 2014; 37(3):389–405. https://doi.org/10.1177/0160017612452429

DSDM:

It can also be used to test the existence of endogenous and exogenous interaction effects in the short term along with the long term [10]. Equation (8) presents this model.

|  | $L\text{nY}_{it}\text{=}\alpha\sum_{j=1}^{n} W_{ij}L\text{nY}_{i,t-1}+\rho\sum_{j=1}^{n} W_{\text{ij}}LnY_{it}+\beta LnX_{it}++\sum_{j=1}^{n} \theta W_{\text{ij}}LnX_{it}+\text{u}_{i}\text{+}\nu_{\text{t}}+\varepsilon_{it}$ | (8) |
| --- | --- | --- |

Equations 9-14 show the direct, spillover, and total effects of the DSDM in the short-term and long-term scales:

|  | $S\text{hort-term direct effects=}\left[\left(I_{N}-\rho W\right)^{-1}\left(\beta_{k}I_{N}+\theta_{\text{k}}W\right)\right]^{\overline{d}}$ | (9) |
| --- | --- | --- |
|  | $S\text{hort-term }spillover\text{ effects=}\left[\left(I_{N}-\rho W\right)^{-1}\left(\beta_{k}I_{N}+\theta_{\text{k}}W\right)\right]^{\overline{\text{rsum}}}$ | (10) |
|  |  | (11) |
|  | $\text{Long-term direct effects=}\left[\left(I_{N}-\rho W-\alpha W\right)^{-1}\left(\beta_{k}I_{N}+\theta_{\text{k}}W\right)\right]^{\overline{d}}$ | (12) |
|  | $\text{Long-term }spillover\text{ effects=}\left[\left(I_{N}-\rho W-\alpha W\right)^{-1}\left(\beta_{k}I_{N}+\theta_{\text{k}}W\right)\right]^{\overline{rsum}}$ | (13) |
|  | $\text{Long-term total effects=}\left[\left(I_{N}-\rho W-\alpha W\right)^{-1}\left(\beta_{k}I_{N}+\theta_{\text{k}}W\right)\right]^{\overline{d}}+\left[\left(I_{N}-\rho W-\alpha W\right)^{-1}\left(\beta_{k}I_{N}+\theta_{\text{k}}W\right)\right]^{\overline{rsum}}$ | (14) |

***Comment 4:*** Discuss the implications of your findings: In the discussion section, it would be valuable to discuss the practical implications of your findings. How can policymakers use the insights from your study to inform their decision-making? What are the potential implications for educational policies and regional development strategies? Providing a clear discussion of the implications will enhance the relevance and impact of your research.

***Response 4:*** Thank you for your comments. Inspired by the reviewers' comments, we provide a clear discussion of the implications in the Discussion section. We further clarify the implications of our findings for educational policies and regional development strategies to help policymakers make decisions more effectively. As mentioned in Section 5 of the revised manuscript (Lines 490-495, page 27):

The findings are of great value to informing policymakers to make educational policies and regional development strategies more effectively consider the short- and long-term economic impacts of LGHEs, regional inequalities in LGHEs and the spatial effects of neighboring regions, which can further promote fair investments in local general higher education, reduce regional disparities, and drive sustainable regional economic growth.

**References:**

8. Akita, T. Educational expansion and the role of education in expenditure inequality in Indonesia since the 1997 financial crisis. Social Indicators Research. 2017; 130(3):1165–1186. https://doi.org/10.1007/s11205-015-1208-y
9. Varughese AR, Bairagya I. Interstate variation in household spending on education in India: Does it influence educational status? Structural Change and Economic Dynamics. 2021; 59:405–415. <https://doi.org/10.1016/j.strueco.2021.09.015>
10. LeSage JP, Pace RK. Introduction of spatial econometrics. 1st ed. New York: Chapman and Hall; 2009.
11. Ojha VP, Ghosh J, Pradhan BK. The role of public expenditure on secondary and higher education for achieving inclusive growth in India. Metroeconomica. 2022; 73(1):49–77. https://doi.org/10.1111/meca.12353
12. Zhu TT, Peng HR, Zhang YJ. The influence of higher education development on economic growth: Evidence from central China. Higher Education Policy. 2018; 31(2):139–157. <https://doi.org/10.1057/s41307-017-0047-7>
13. Anselin L. Thirty years of spatial econometrics. Papers in Regional Science. 2010; 89(1):3–25. https://doi.org/10.1111/j.1435-5957.2010.00279.x
32. Bao ZY, Huang DF. Gender differences in reactions to enforcement mechanisms: A large-scale natural field experiment; 2020 [cited 2023 Sep 26]. Database: SSRN [Internet]. Available from: http://dx.doi.org/10.2139/ssrn.3641282
33. Chen MZ, Huang DF, Wu BY. Interlocking Directorates and Firm Performance: Evidence from China; 2022 [cited 2023 Sep 26]. Database: SSRN [Internet]. Available from: <http://dx.doi.org/10.2139/ssrn.4005022>
52. Elhorst JP. Matlab Software for Spatial Panels. International Regional Science Review. 2014; 37(3):389–405. https://doi.org/10.1177/0160017612452429

*We earnestly appreciate your work, and we thank you for your valuable comments.*

*We tried our best to improve the original manuscript and made significant changes in the revised manuscript. These changes will not influence the content of the article. Here, we earnestly appreciate the reviewer’s work and hope that the revised version will meet your approval. In the meantime, we appreciate the editor’s invitation to modify our manuscript. Thank you for giving us the chance to submit the revised manuscript.*

*Once again, thank you very much for your comments and suggestions.*

*Best wishes,*

*Authors of this article*
